# 

**Published:** 1901-12-07

**Authors:** 


### NOTICE TO CORRESPONDENTS.

All MSS., Letters, Books for Review, and other matters intended for the Editor should be addressed The Editor, "The Hospital," The Hospital Building, 28 & 29 Southampton Street, Strand, W.C.

The Editor cannot undertake to return rejected MS., even when accompanied by stamped directed envelope.

**Notice to Advertisers and Subscribers.**

All Advertisements, Orders for Copies of The Hospital, and Business Communications should be addressed to **THE MANAGER (Not to the Editor),** The Hospital, 28 & 29 Southampton Street, Strand, London, W.C.

The Cost of Subscription, post free, is as follows:—Per week, 2½d.; per quarter, 2s. 8d.; per half-year, 5s. 3d.; per annum, 10s. 6d. Foreign:—Per annum, 15s. 2d., payable, in all cases, in advance.

**NOTICE.—Vol. XXX. of "The Hospital" (April 1901, to September 1901), in handsome cloth binding can now be obtained at the office of this Journal, price, 7s. 6d. Cases for binding the Numbers contained in this Volume 1s. 6d. Index to Vol. XXX., 2½d., post free.**

**The Monthly Part for December is now ready. Price 6d., by post 8d.**

### SCRAPS AND GLEANINGS.

A dance in aid of the Charing Cross Hospital is to be held at the Empress Rooms, Royal Palace Hotel, on January 7, 1902.

The annual exhibition of work done by the London Needlework Guild took place at the Imperial Institute recently. The total number of articles sent in is about 60,000; last year the contributions amounted to 61,517.

The foundation stone of the new out-patients' department of the Dover Hospital, which is being erected as a memorial to the late Queen, was laid by Lady Pearson recently. An operating theatre is also being built as the town's memorial to the late Sir R. Dickeson.

### THE STUDENT OF MEDICINE.

### APPOINTMENTS.

I. Aird, M.B., B.S.Edin., appinted Certifying Factory Surgeon for the Bangor District of County Down. **A. J. M. Armstrong,** L.R.C.P.Lond., M.R.C.S.Eng., appointed Medical Officer for the Southgate District of the Edmonton Union. **Arthur Cant,** M.B., B.Ch. (Birmingham University), M.R.C.S., L.R.C.P.Lond., appointed Medical Officer to the Marston Green Cottage Homes, Medical Officer for the Coleshill and Maxstoke Districts of the Menden Union, and Public Vaccinator for Coleshill, Warwickshire.

**W. H. Cuthbert**, L.R.C.P., L.R.C.S.Edin., appointed Medical Officer of Health to the Frinton-on-Sea Urban District Council. **Thomas Evans**, M.B., appointed Medical Officer to the Holborn Union Workhouse. **J. Percy Good**, M.B., Ch.B.Vict., appointed District Medical Officer to the Pendleton Branch Dispensary of the Salford Hospital. **F. W. Halliday**, M.R.C.S., L.R.C.P.Lond., appointed Surgeon for the D Division of the Leeds City Police. **G. H. Heald**, L.R.C.P.Edin., M.R.C.S.Eng., appointed Surgeon for the A Division of the Leeds City Police. **J. W. Hutton**, M.R.C.S., L.R.C.P.Lond., appointed Surgeon for the C Division of the Leeds City Police. **C. D. Ingle**, M.R.C.S., L.R.C.P.Lond., appointed Certifying Factory Surgeon for the Somerton Distric of the County of Somerset, **R. J. Johnstone**, B.A., M.B., B.Ch., B.A.O.R.U.I., appointed Assistant to the Gynæcologist o tthe Royal Victoria Hospital, Belfast. **E. Kennington**, M.C.C S., L.R.C.P. Lond., appointed Medical Officer of the Aylsham Union. **Sydney H. Long**, M.B.Cantab., appointed Physician to the Jenny Lind Infirmary for Sick Children, Norwich. **H. J. Monypeny**, M.B., B.Ch., B.A.O.R.U.I., appointed Honorary Anæsthetist to the Royal Victoria Hospital, Belfast. **R. Muschamp**, L R.C P., L.R.C.S.Edin., appointed Medical Officer of Health for the Yeadon Urban District. **L. L. Preston**, M.B., B.S.Durh., appointed Medtcal Officer of Health to the St. Helens Urban District Council. **A. B. Reid**, M R.C.S., L.R.C.P., appointed House Surgeon to the Paddington Green Children's Hospital. **R. Watson Reid**, M B, Ch.B., appointed House Physician to the Paddington Green Children's Hospital. **J. D. Richmond**, M.B., B.S.Glasg., appointed Assistant to the West Derby Union Infirmary. **J. W. S. Seccombe**, M.R.C.S., L.R.C.P., appointed Junior House Surgeon to the Radcliffe Infirmary, Oxford. **P. J. A. Seccombe**, M.B., B.C.Camb., appointed District Medical Officer of the Uxbridge Union. **Norman J. Sinclair**, M.B., Ch.B.Aberd., appointed Medical Officer of Health for the Burgh of Leith, and Police Surgeon. **H. de Carle Woodcock**, L.R.C.P., L.R.C.S. Edin., appointed Surgeon for the B Division of the Leeds City Police.

St. Thomas's Hospital.—House appointments.—The following gentlemen have been selected as House Officers from Tuesday, December 3rd, 1901:—House Physicians: **L. H. C. Birkbeck**, B.A., M.B. B.Ch.Oxon. (*Extension*); **V. S. Hodson**, B.A., M.B. B.Ch.Oxon. (*Extension*); **W. M. G. Glanville**, B.A., M.B., B.Ch.Oxon.; **T. W. S. Paterson**, M.A., M.B., B.C.Cantab., L.R.C.P., M.R.C.S. Assistant House Physicians: **T. B. Henderson**, M.A., M.B., B.Ch.Oxon; **H. S. Stannus**, L.R.C.P., M.R.C.S. House Surgeons (*Extension*): **C. A. R. Nitch**, M.B.Lond, L.R.C.P., M.R.C.S.; **W. H. O. Woods**, B.A., M.B., B.C.Cantab.; **S. Hunt**, L.R.C.P., M.R.C.S.; **A. S. Grimwade**, L.R.C.P., M.R.C.S. Assistant House Surgeons (*Extension*): **G. A. C. Shipman**, M.A., M.B., B.C.Cantab., L.R.C.P., M.R.C.S.; **W. Hill**, B.A.Cantab., L.R.C.P., M.R.C.S.; **T. W. H. Downes**, L.R.C.P., M.R.C.S.; **F. J. Child**, M.A., M.B., B.C.Cantab., L.R.C.P., M.R.C.S. Obstetric House Physicians: (*Senior*) **Z. Mennell**, M.B.Lond., L.R.C.P., M.R.C.S.; (*Junior*) **H. T. D. Acland**, L.R.C.P., M.R.C.S. Clinical Assistants: (Throat) **A. D. Hamilton**, L.R.C.P., M.R.C.S. (*Extension*); **A. P. Bowdler**, B.A.Cantab., L.R.C.P., M.R.C.S. (Skin) **F. Clarkson**, M.B., B.S.Durh. (*Extension*); **J. L. Timmins**, M.A., B.C.Cantab., L.R.C.P., M.R.C.S. (*Extension*). (Ear) **F. Clarkson**, M.B., B.S.Durh. (*Extension*). (Electrical Department) **L. S. Dudgeon**, M.R.C.P, M.R.C.S.

